# Long Lasting Local and Systemic Inflammation after Cerebral Hypoxic ischemia in Newborn Mice

**DOI:** 10.1371/journal.pone.0036422

**Published:** 2012-05-02

**Authors:** Max Winerdal, Malin Elisabeth Winerdal, Johan Kinn, Vijay Urmaliya, Ola Winqvist, Ulrika Ådén

**Affiliations:** 1 Department of Woman and Child Health, Karolinska Institutet, Stockholm, Sweden; 2 Department of Medicine, Unit of Translational Immunology, Karolinska Institutet, Stockholm, Sweden; 3 Department of Physiology and Pharmacology, Karolinska Institutet, Stockholm, Sweden; Hôpital Robert Debré, France

## Abstract

**Background:**

Hypoxic ischemia (HI) is an important cause of neonatal brain injury and subsequent inflammation affects neurological outcome. In this study we performed investigations of systemic and local activation states of inflammatory cells from innate and adaptive immunity at different time points after neonatal HI brain injury in mice.

**Methodology/Principal Findings:**

We developed a multiplex flow cytometry based method combined with immunohistochemistry to investigate cellular immune responses in the brain 24 h to 7 months after HI brain injury. In addition, functional studies of *ex vivo* splenocytes after cerebral hypoxic ischemia were performed. Both central and peripheral activation of CD11b^+^ and CD11c^+^ antigen presenting cells were seen with expression of the costimulatory molecule CD86 and MHC-II, indicating active antigen presentation in the damaged hemisphere and in the spleen. After one week, naïve CD45rb^+^ T-lymphocytes were demonstrated in the damaged brain hemisphere. In a second phase after three months, pronounced activation of CD45rb^−^ T-lymphocytes expressing CD69 and CD25 was seen in the damaged hemisphere. Brain homogenate induced proliferation in splenocytes after HI but not in controls.

**Conclusions/Significance:**

Our findings demonstrate activation of both local and systemic immune responses months after hypoxic ischemic neonatal brain injury. The long term immune activation observed is of general importance for future studies of the inflammatory response after brain injury as most previous studies have focused on the first few weeks after damage, while the effects of the late inflammation phase may be missed. Furthermore, the self-reactive component raises the question if there is a correlation with development of autoimmune brain disease later in life.

## Introduction

The intricate interactions of innate and adaptive immunity play a major part in the pathology of many inflammatory conditions and inflammation has been identified as a key factor for neurological outcome after asphyxia. [1] Hypoxic ischemia (HI) after asphyxia is an important cause of neonatal brain injury and the incidence of moderate to severe hypoxic ischemic encephalophathy is 0.5–2 per 1000 live births in the developed world [2] and much more common in low income countries. [3]

Studies during the last two decades have shown that immune cells are involved in the pathogenesis of reperfusion damage in the brain after HI [1] [4] and the innate immune mechanisms with cytokine effects and subsequent activation of microglia, granulocytes and other innate cells have been extensively examined. [5] Interestingly, a recent report describes activated dendritic antigen presenting cells [6], as a possible link to adaptive immunity after brain injury. Local T-cell infiltration has been reported in rodent models after HI in neonates [7] and stroke in adults [8], but data of their functional status and activation state is missing. However, there is circumstantial evidence for a functional role of lymphocytes: Antibody blocking of the Very Late Antigen-4 (VLA-4), known to block lymphocyte migration into inflamed tissue, is protective 72 h after adult HI [9], and T- and B-cell depleted animals have reduced infarctions 22 h after stroke in adult animals. [10] Furthermore, depletion of regulatory T-cells, a subpopulation with anti inflammatory properties, increases the brain injury after ischemia [11]. These findings suggest that there is a short term detrimental effect of adaptive immune cells, but the long term effect is still entirely unknown. Interestingly, it was recently shown in humans that Th1 primed activated T-cells were present in peripheral blood after stroke [12] and elevated levels of antibodies against neuroantigens have also been observed in stroke patients [13], [14], which suggest induction of adaptive immune responses.

To better understand the complex interplay between innate and adaptive immunity after brain injury, we set out to investigate the temporal dynamics of key immune cell populations. Markers for principal cellular events such as antigen presentation, activation and memory T-lymphocyte characteristics were also studied in the brain and peripherally in the spleen. Immune cells were characterized by multiplex flow cytometry, immunohistochemistry and proliferation assays. Whereas previous reports have focused on the early infiltration and inflammatory response [15], the plausible participation of adaptive immunity implies long term effects, why the cellular pattern was studied up to seven months after the brain injury.

## Materials and Methods

This study and all experiments were approved by the local ethics committee, Stockholm norra djurförsöksetiska nämd, ethical permit number N152/09 and carried out in accordance with local institutional guidelines.

### Animals

C57/bl6 mice (SPF) with free access to pelleted food were used in the experiments. Pups were kept with dam at all times except the actual surgery and hypoxia. Animals were euthanized by injection of 240 mg/kg sodium pentobarbiturate *i.p.* and the tissue was percoronarly perfused with 12 mL phosphate buffered saline (PBS) to remove intravascular blood cells.

### Hypoxic ischemic brain injury

We used a modified version of the Vannucci model [16] as described previously [17] with unilateral electrocoagulation at 8W of the right carotid artery via midline neck incision under isoflourane sedation and local bupivacaine. The pups rested with the dam 1 h after the operation, followed by 1 h of 10% O_2_ in 90% N_2_ at 36°C±1°C skin temperature.

### Immunohistochemistry

HI and sham operation was performed at postnatal day 10. Brains were collected after HI at day 11 (n = 3), day 12 (n = 3), day 17 (n = 3) and day 100 (n = 3). Sham operated controls were analyzed at day 17 (n = 5). In total 17 animals were used for immunohistochemistry. Three levels of each brain were collected, corresponding to bregma 1.32 mm, −1.64 mm and −2.92 mm in the adult mouse brain using cryostat 10–12 µm sections. The tissues were fixed for 10 min in 4% paraformaldehyde. Endogenous peroxidase was blocked by 0.3% H_2_O_2_ in 30% Normal Horse Serum for 10 min and tissues incubated with antibodies against VCAM-1, CD3 (BD Biosciences) or MAP-2 (Sigma). For MAP-2 staining VECTOR® M.O.M.™ Immunodetection Kit and for CD3 and VCAM-1 stainings VECTASTAIN® Elite ABC-Peroxidase Kit were used according to the manufacturers' specifications. Pictures were recorded with a Nikon eclipse E800 light microscope with an Olympus DP70 camera with DP controller, version 3.1.1.267 acquisition software. Percent stained area in each hemisphere of brain slice was evaluated using Image J, version 1.42 n.

### FACS Analysis

**Figure 1:**
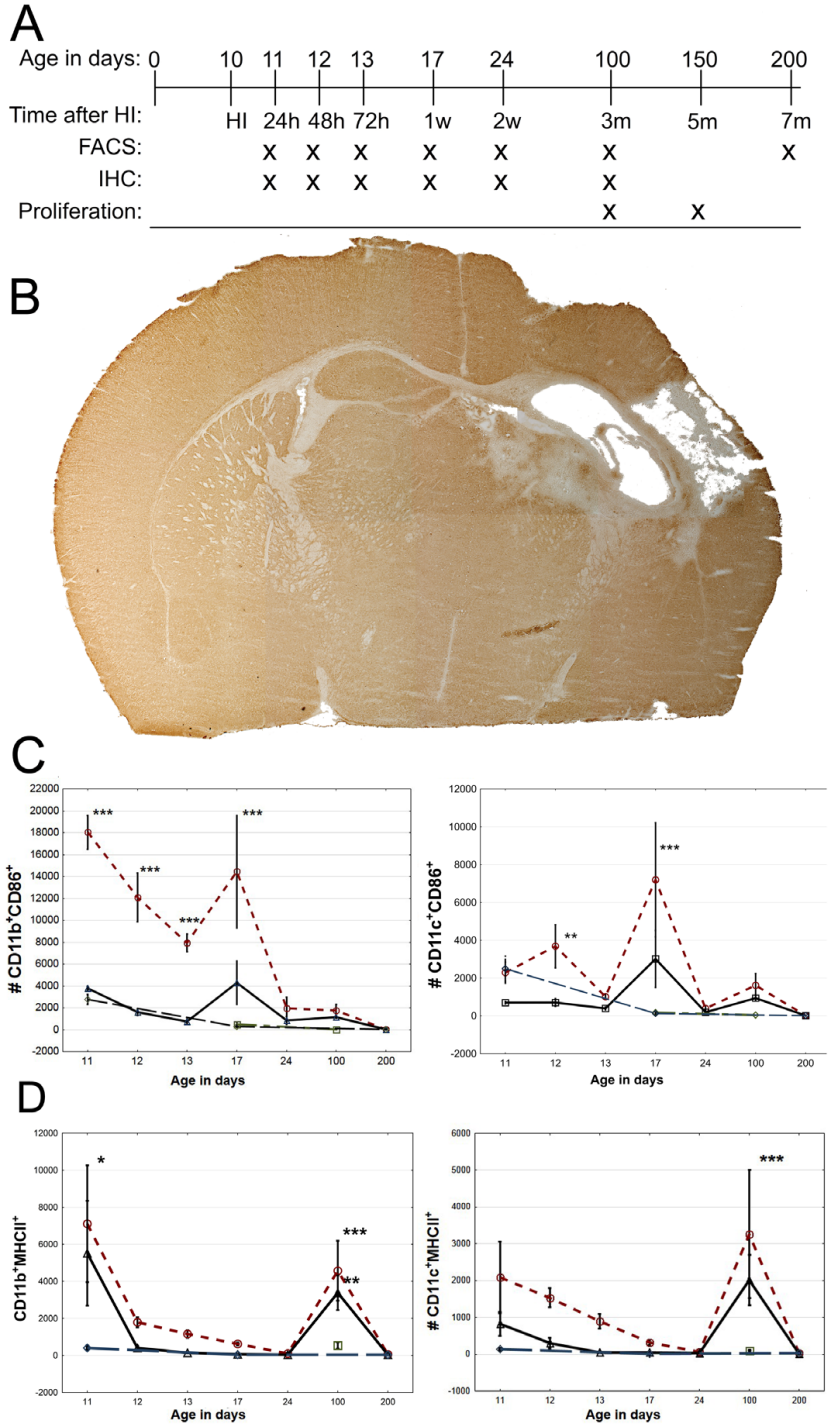
Local innate inflammatory cell response after hypoxic ischemic (HI) brain injury. (A) Time-line with experimental setup. (B) Representative cross-section of MAP-2 (live neurons) stained mouse brain two weeks after brain damage. (C) Number of CD11b^+^ (left) and CD11c^+^ cells (right) expressing the activation marker CD86 over time. (D) Number of CD11b^+^ (left) and CD11c^+^ cells (right) expressing MHC-II (H2IAb) over time. Red circle = infarcted hemisphere, black triangle = uninjured hemisphere, blue diamond = sham operated, and green square = unoperated. * = p<0.05, ** = p<0.01 and *** = p<0.001. Compared to sham and unoperated controls. Error bars represent SEM.

HI was performed at postnatal day 10 and samples were analyzed at day 11 (n = 6), day 12 (n = 7), day 13 (n = 7), day 17 (n = 6), day 24 (n = 6), day 100 (n = 7) and day 200 (n = 3). Sham operated controls were analyzed day 11 (n = 3), day 17 (n = 3) and day 200 (n = 5). Unoperated animals were analyzed at day 17 (n = 6) and day 100 (n = 3). For a schematic time-line see Figure 1A. In total 62 animals were used for FACS analysis. Statistical power analysis was performed on pilot data (n = 3 per group) 1 week after HI, not included in further analyses in this study. Cells from the damaged and undamaged brain hemispheres and spleen were prepared as follows: Brains were collected and prefrontal part and cerebellum were removed. The spleen, damaged and undamaged hemisphere of the brain were put in ice-cold RPMI-1640 medium. Samples were homogenized with a glass homogenizer and filtered through a 100 µm celltainer filter. Spleen cell suspensions were hemolyzed with ACK-buffer (0.15 mol/L NH_4_Cl, 1 mmol/L KHCO_3_, 0.1 mmol/L EDTA and H_2_O, pH 7.2) for 5 min and washed in PBS. Samples were spun at 300×g, cells incubated with anti-CD16/32 Fc-receptor block for 10 min and washed in FACS buffer (2% BGS, 0.1% NaN_3_ in PBS) before surface staining. In this study we omitted any density gradient or enzymatic digestion steps used in other studies [8], [18] to avoid any selection bias, loss of cells, modification to surface marker expression or cell function that has previously been reported in comparative methodological studies [19], [20], [21].

Samples were stained with antibodies, for details on markers and target cell populations see Methods S1 (table S1); TCR-β APC-Cy7 and I-Ab PE from Biolegend, as well as Ly-6G FITC, CD8a PerCP-Cy5.5, CD86 APC, CD11b APC-Cy7, CD11c PE-Cy7, CD4 APC, CD45rb PE from BD Pharmingen, and DAPI. Flow cytometric data was collected on a FACSAria (BD Biosciences) and analyzed using the FACSDiva software. Gates were set by investigators blinded to treatment and time point. Approximately 2.5×10^6^ events were analyzed from each brain hemisphere and 1×10^6^ cells from each spleen, reported as positive per million live cells (DAPI^−^). The cell content after HI brain injury was compared to sham or unoperated controls. For FACS analysis, gates were set to filter out debris in forward and side scatter, with a negative DAPI gate to exclude dead cells. Gates were set on histograms from positive and negative controls for each separate experiment. The same gates were used on all samples run simultaneously on both spleen and brain, including controls and operated animals when applicable.

### Proliferation assay

Thymidine incorporation based proliferation assays were performed as previously described [22], using brain homogenate as stimuli and PBS as negative control n = 4 for each group. Single-cell suspensions of splenocytes were prepared from unoperated, age-matched controls 3 and 5 months after HI as described for flow cytometry, and dissolved in RPMI-1640 with 10% BGS, 1% PeSt, 1% Glutamine, 50 µM 2-ME, and 1% Pyruvate. 3–4×10^5^ cells per well were added to 96-well plates and stimulated with brain homogenate or PBS. Brain homogenate was prepared from damaged/undamaged brain, homogenized in 5 mL RPMI-1640, and kept at −20°C. For stimulation, brain homogenate was added at a ratio of 1∶100. Plates were kept at 37°C for two to six days, pulsed with 1 µCi/well [^3^H]Thymidine for 18 h and frozen at −20°C. Plates were thawed and well content transferred to a glass fibre filter (Wallac) by a cell harvester (TOMTEC). For detection of radioactivity, Meltilex A – Melt on scintillation sheets (Wallac) were used and radioactivity was measured in a 1205 Betaplate Liquid Scintillation Counter (Wallac). Proliferation index was calculated as: cpm(brain homogenate)/cpm(PBS).

### Statistical analysis

All statistical analysis used Statistica 8.0 from Statsoft. Inc. Factorial Anova was used with Tukey or Dunnett post-hoc test when appropriate.

## Results

To better understand inflammation after HI in the neonatal brain, we investigated different immune cell populations from innate and adaptive immunity after acute unilateral infarction in the immature brain. No significant differences were seen between sham operated animals (24 h and 1 week after the skin incision) and age matched controls, indicating that brain immune cell content can be regarded as relatively stable during unchallenged conditions, unaffected by sham operation. Therefore both unoperated and sham operated animals were used as baseline controls to calculate p-values, the groups and SEM are however presented separately in the graphs. Loss of MAP-2 staining by immunohistochemistry was used to visualize a typical lesion (Figure 1B).

### Immune response in the brain

We developed a multiplex flow cytometry based method for investigation of cellular immune responses after brain HI. To minimize the influence from *ex vivo* procedures, cells were promptly analyzed without any further purification steps or procedures except immunostaining. For reproducible results; debris and aggregates were filtered out, DAPI used to exclude dead cells, a high number of events collected and a strict gating strategy applied. Known splenetic cell populations were used as reference to validate the gating strategy in brain.

Initially there was a rapid activation of innate CD11b^+^ cells as judged by upregulation of the costimulatory molecule CD86 (B7.2) in the injured hemisphere after HI (Figure 1C). The initial activation wave at 24 h was followed by a second increase of activated CD11b^+^CD86^+^ cells, with the maximum peak at one week after the insult, succeeded by a return to normal levels after two weeks (Figure 1C). The number of CD11c^+^ cells and Ly-6G^+^ granulocytes increased in both hemispheres 24 to 48 hours after HI (data not shown). However, the activation of CD11c^+^ cells increased significantly after 48 h only in the damaged hemisphere, followed by a second wave of activated CD11c^+^CD86^+^ cells observed after one week (Figure 1C). MHC-II expression in CD11b^+^ cells increased 24 h after injury, returned to normal levels after 48 h and later increased again after three months (Figure 1D). In contrast, CD11c^+^ cells first displayed significant upregulation of MHC-II at three months after brain damage (Figure 1D).

**Figure 2:**
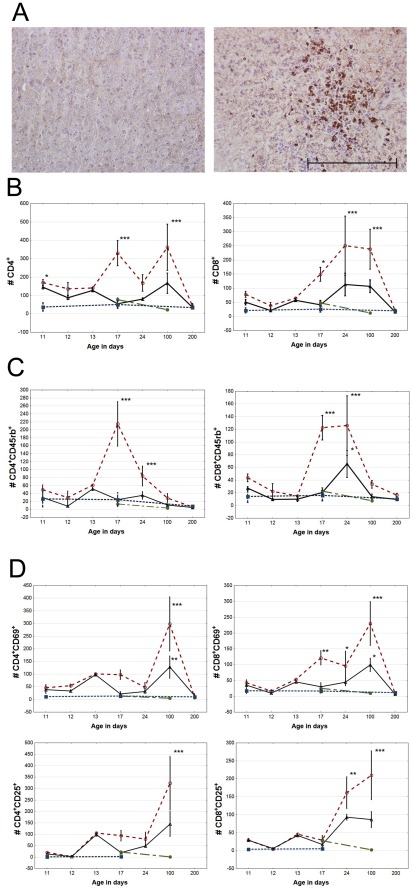
Local T-cell response after hypoxic ischemic (HI) brain injury. (A) T-lymphocytes infiltrating injured (right) and uninjured (left) hemisphere, visualized by anti-CD3 immunohistochemistry. Ruler indicates 200 µm. (B) CD4^+^ (left) and CD8^+^ (right) T-lymphocyte infiltrating cells per 10^6^ live cells after HI brain injury (C) the expression of the naïvety marker CD45rb, and (D) the activation markers CD69 and CD25 in these populations. Open red circle = infarcted hemisphere, black triangle = uninjured hemisphere, blue square = sham operated and green filled circle = unoperated.

We also investigated the activation and recruitment of T-cells. Initially, sections from mice with HI brain injury were stained with an antibody against the pan T-cell marker CD3. As demonstrated in Figure 2A, the unaffected hemisphere contained no detectable infiltrates two weeks after the injury, whereas the infarcted area contained substantial infiltrates of CD3^+^ T-lymphocytes. Subsequently, we performed temporal FACS analysis of T-lymphocyte influx into both hemispheres after HI brain injury on individual mice (Figure 2B). The initial influx of CD4^+^ T-lymphocytes peaked one week after HI with a more than three-fold increase compared to baseline. Interestingly, the initial CD4^+^ influx into the affected hemisphere was followed by recruitment of CD8^+^ cytotoxic T-cells, peaking two weeks after HI.

The T-cells entering the injured hemisphere after one week expressed the naïve T-cell marker [23] CD45rb (Figure 2C). To our surprise, we found a significant number of naïve CD8^+^ T-lymphocytes entered the undamaged hemisphere after two weeks, whereas no entrance of naïve CD4^+^ T-cells was demonstrated in the undamaged hemisphere (Figure 2C, left). The number of naïve CD45rb expressing CD4^+^ and CD8^+^ cells returned to normal within three months.

**Figure 3:**
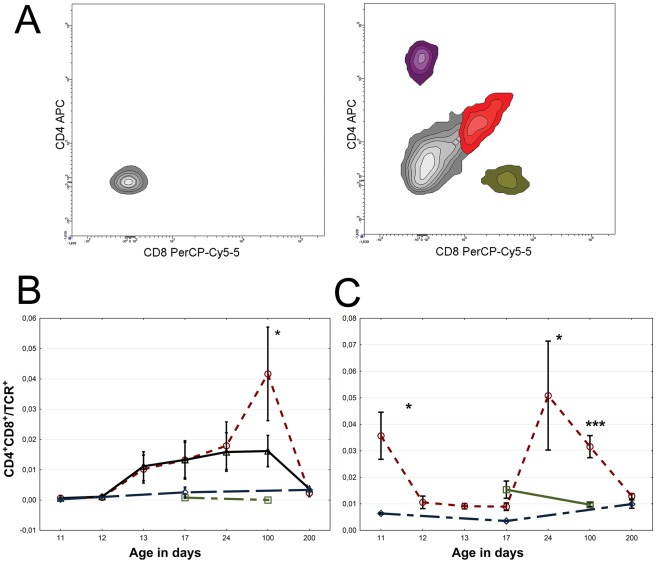
Presence of double-positive CD4^+^CD8^+^ T-cells after hypoxic ischemic brain injury. (A) CD4^+^ and CD8^+^ T-lymphocytes in unoperated age matched control (left) and damaged brain hemisphere (right) two weeks after HI. Note the double positive population marked in red. (B) Proportion CD4^+^CD8^+^ double positive T-lymphocytes in brain and (C) spleen after HI. Red circle = infarcted hemisphere (B) or HI (C), black triangle = uninjured hemisphere, blue diamond = sham operated, and green square = unoperated.

In addition, we stained T-lymphocytes for expression of the very early activation marker [24] CD69 and the IL-2 receptor α-chain (CD25). The expression of CD69 and CD25 increased both in CD4^+^ and CD8^+^ populations reaching maximum levels three months after HI brain injury (Figure 2D). A subpopulation of CD4^+^CD8^+^ double positive T-cells, not seen in controls, appeared in the damaged brain at three months (Figure 3A and 3B), but disappeared again at seven months. Furthermore, the T-cells which infiltrated the brain three months after HI were mainly CD4^+^CD45rb^low^ (Figure 2B, C).

### Immune response in the spleen

**Figure 4:**
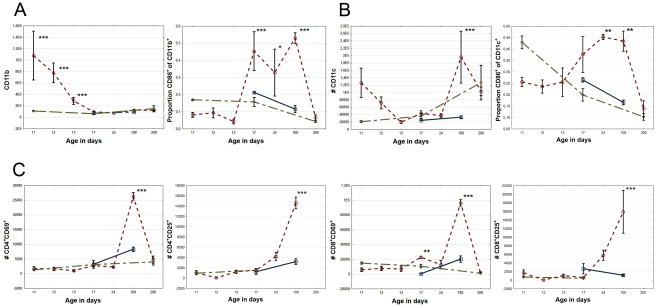
Systemic inflammatory cell response after hypoxic ischemic (HI) brain injury. (A) CD11b^+^ cells per 10^6^ live cells in the spleen over time after HI brain injury (left) and the CD86^+^ proportion of these cells (right). (B) CD11c^+^ cells per 10^6^ live cells (left) and the CD86^+^ proportion of these cells (right). (C) CD4^+^ and CD8^+^ T-lymphocytes expressing the activation markers CD69 and CD25 in spleen. Red circle = HI, green diamond = sham operated, and blue square = unoperated.

In parallel, we investigated the immune response in the periphery by flow cytometry of splenocytes from HI brain injured animals. Firstly, the HI induced peripheral effects on splenic macrophages and dendritic cells were investigated. There was an initial rapid ten-fold increase in number of CD11b expressing cells (Figure 4A) accompanied by an five-fold increase in Ly-6G^+^ granulocytes (data not shown), already after 24 h. These populations returned to normal after one week. The initial increase in CD11b expressing cells was followed by an activation phase after one week as determined by the increased expression of the costimulatory molecule CD86 (Figure 4A, right). Initially, there were no significant differences in the number of CD11c^+^ cells in the spleen after HI brain damage, but at three months a transitory increase was observed (Figure 4B). In addition, there was an increase in the proportion activated CD86 expressing CD11c^+^ dendritic cells from one week to three months after injury (Figure 4B, right).

We next set out to investigate if the increased activation of peripheral antigen presenting cells in the spleen after HI brain injury resulted in activation of the adaptive immune system. The HI injured mice demonstrated a brief activation of CD8^+^ T-lymphocytes in the spleen at one week measured by a significant increase in the very early activation marker CD69 expression (Figure 4C). At three months after induced brain injury, a second more pronounced activation peak of both CD4^+^ and CD8^+^ T-lymphocytes was observed (Figure 4C). Notably, a CD4^+^CD8^+^ double positive population first appeared briefly after 24 h, returned at two weeks and remained significantly increased up to three months after brain HI (Figure 3C).

### Functional status

**Figure 5:**
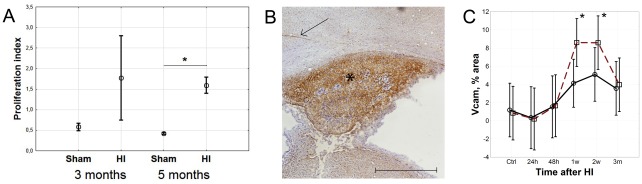
Splenocyte proliferation and VCAM-1 expression in brain after hypoxic ischemic brain injury. (A) Proliferation index of splenocytes from mice three and five months after HI or sham operation measured by [^3^H] Thymidine incorporation after stimulation with brain extract. (B) VCAM-1 expression in the infarcted hippocampus region (star) and blood vessels (arrow) as visualized by immunohistochemistry. Ruler indicates 500 µm. (C) VCAM-1 expression in damaged (squares) and undamaged (circles) hemispheres over time after HI brain injury.

To clarify the implications of the peripheral activation of splenocytes after HI brain injury, we performed functional stimulation tests. Recall assays three and five months after injury, using brain from extract as stimulus revealed an almost four-fold increase in proliferation index of splenic T-cells from brain injured compared to sham operated mice (Figure 5A). The proliferation was increased to a similar extent when homogenate from infarcted hemisphere, undamaged hemisphere, unoperated control brain or spinal cord was used (data not shown).

### Homing to the brain

Next, we investigated a possible mechanism of T-lymphocyte homing to the brain by immunohistochemistry using antibodies against Vascular Cell Adhesion Molecule 1 (VCAM-1). Increased VCAM-1 expression was observed in the infarcted area of the injured brain hemisphere and associated blood vessels (Figure 5B), to a significant degree from one to two weeks after injury (Figure 5C) with a tendency towards increased expression after three months. In addition, there was a slight increase of VCAM-1 expression in the non-injured brain hemisphere, although not significant (Figure 5B).

## Discussion

Using multiplex flow cytometry we describe key events in cellular immune infiltration and activation after aseptic brain hypoxic ischemia in neonate mice. We visualized, both in the post ischemic brain and systemically, the infiltration and activation of innate antigen presenting cells and for the first time, the sequential activation of T-lymphocytes. The flow cytometric data are supported by *ex vivo* functional data demonstrating self-reactivity with a lymphocyte response stimulated by brain antigens five months after HI brain injury.

### Activation of antigen presenting cells

The first immune activation observed after HI consisted of brain CD11b^+^ cells and has previously been recognized as foremost microglia, in contrast to the second activation peak after one week which mainly consists of infiltrating macrophages. [25] The early activation of CD11c^+^CD86^+^ cells is also in agreement with earlier reports. [6] Interestingly, CD11b^+^ microglial cells significantly increased their MHC-II expression after 24 h, and cerebral CD11b^+^ and CD11c^+^ cells three months after HI (Figure 1D), thereby allowing interactions between APCs and T-cells.

A recent report by Hug et al describes reduced stimulatory capacity of circulating APCs three days after injury in an adult stroke model [26]. Interestingly, these results correspond well with our finding that peripheral APCs show a tendency toward reduced CD86 expression compared to controls during the first few days after HI (Figure 4A–B). Notably however, starting one week after HI, an extensive increase in the proportion of CD86 expressing CD11b^+^ and CD11c^+^ cells was seen which persisted at three months but had returned to normal seven months after HI (Figure 4A–B). Microglial cells have a common progenitor with monocytes and share CD11b and CD11c markers [27]. Our results indicate that brain HI activates antigen presenting cells originating from the brain and bone marrow. Furthermore, our data clearly demonstrates systemic activation of antigen presenting cells (APCs) alongside the local brain responses.

### Induction of adaptive immunity

The increase of CD86^+^ and MHC-II expressing APCs in the brain after HI suggests preparation for activation of adaptive immune responses. In adult stroke models, T-lymphocytes have been increasingly implicated in the pathophysiology following stroke [28], [29] and it is apparent that the local inflammatory response is accompanied by massive systemic effects [30]. We therefore investigated recruitment and activation of T-lymphocytes. Although there have been earlier reports of T-cell infiltration, hitherto the functional status of infiltrating lymphocytes after brain injury has been unknown and there has been some controversy in the literature regarding what population of T-lymphocytes dominate after brain injury [7], [31]. Our data demonstrates that the initial influx of T-lymphocytes was dominated by CD4^+^ T-helper cells, followed one week later by CD8^+^ cytotoxic T-cells (Figure 2A), where the temporal profiles could explain the previous discrepancy.

The significant upregulation of VCAM-1 in the damaged hemisphere one week after the insult (Figure 5B and 5C) suggests recruitment of lymphocytes. VCAM/VLA-4 interactions have been demonstrated to play a major role in lymphocyte trafficking to the brain after stroke in adult animals [32]. Strikingly, in our model VCAM-1 expression correlates perfectly in time with the appearance of brain infiltrating T-lymphocytes at one week.

The T-cells entering the injured brain hemisphere one week after HI expressed the naïve T-cell marker CD45rb, but three months after HI few CD45rb^+^ cells were seen (Figure 2C). The diminished number of CD45rb expressing cells could either be due to efflux of lymphocytes or activation and maturation. [23] Since the number of infiltrating lymphocytes is not affected (Figure 2B) the data suggests that the T-cells remain in the brain, become activated and down-regulate the expression of CD45rb. To test this hypothesis we stained T-lymphocytes for the activity markers CD69 and CD25 and could indeed demonstrate a prolonged activation in the brain (Figure 2D). Gelderblom *et al* reported early brain T-cell infiltration up to one week after adult mouse stroke, but found no significant changes in activation status. [8] This is mainly in agreement with our results, where the activation of T-lymphocytes was first detectable one week after HI, with the highest activation levels observed three months after damage. The differences seen could be explained by both the model used and/or the age of animals as well as differences in methodology prior to flow cytometric analysis. To our knowledge this is the first report of activated T-cells in the brain after HI.

### T-lymphocyte characteristics coherent with self reactivity

The activation of T-cells after an aseptic insult raises the question whether the late phase activation is recognizing self, and thus is part of an autoimmune reaction against damaged brain tissue. Intriguingly, the subpopulation of CD4^+^CD8^+^ double positive T-lymphocytes appearing in the damaged brain and spleen post HI (Figure 3), has in the human setting been linked to different autoimmune diseases. [33] In the autoimmune disorder multiple sclerosis (MS), circulating CD4^+^CD8^+^ T-lymphocytes has been found to increase [34], be pathogenic and infiltrate the CNS. [35] Moreover, the T-lymphocytes which infiltrated the brain three months after HI were mainly CD4^+^CD45rb^low^ (Figure 2C), a cell population which infiltrates the CNS in the murine model of MS, experimental allergic encephalomyelitis (EAE) [36]. Activated peripheral T-cells recognizing brain antigens as well as elevated autoantibody levels towards neuroantigens have been observed in humans after stroke [13], [14], [37], and it has been speculated that this may influence the development of stroke related dementia in adults [13]. In clinical data, there is increasing evidence for a relation between fetal/neonatal infection/inflammation and neurodevelopmental impairments such as cerebral palsy. [38] One could therefore speculate that the long term clinical manifestations after brain HI would present as immune mediated neurodevelopmental disorders rather than well-established autoimmune diseases such as MS.

### Functional implications

Interestingly, brain homogenate was able to stimulate proliferation in the post HI splenocytes whereas cells from sham operated animals did not respond. Since brain tissue is not immunogenic during normal conditions the increased proliferation is most likely due to an antigen specific, long-lived peripheral activation of T-cells following brain injury. In this context, the report of activated peripheral T-lymphocytes in humans after stroke is intriguing. [12] Since the cellular immune responses after HI brain injury varied less than the damage size seen in previous studies, the clinical outcome is probably not only dependent on the degree of immune activation but also on which antigens are targeted and the cytokine milieu. The actual antigens involved, clonality and function of the responding T-lymphocytes warrant further investigation.

### Summary

**Figure 6:**
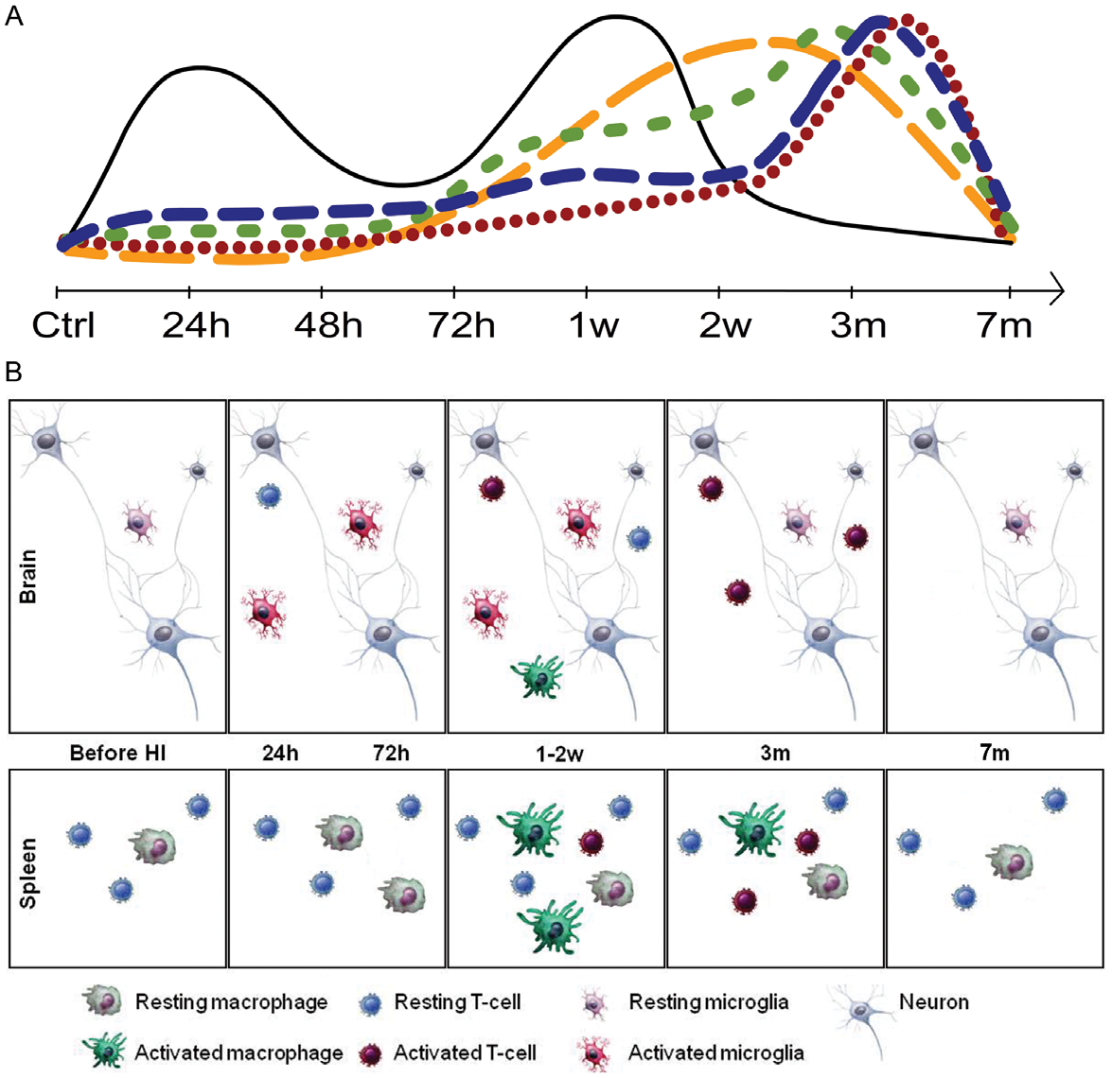
Local and peripheral dynamics of innate and adaptive immune activation after hypoxic ischemic brain injury. (A) Temporal correlation of activated cells after HI brain injury. Note the 24 h and one week activation of CD11b^+^ cells as well as brain T-cell infiltration after one week. Black filled line; activated CD11b^+^CD86^+^ cells in the brain. Green short dashed line; activated CD8^+^CD69^+^ and blue long dashed line; CD4^+^CD69^+^ T-lymphocytes in the brain. Red dotted line; activated splenetic T-lymphocytes. Yellow long dashed line; Proportion CD86 expressing splenetic APCs. (B) Schematic overview of cellular inflammation after HI brain injury.

In conclusion, our results converge to form a dynamic temporal model of both central and peripheral, innate and adaptive immune activation after HI brain injury (Figure 6). The observed downregulation of CD45rb in brain infiltrating cells in combination with an activated phenotype after three months and peripheral responsiveness to brain homogenate is intriguing. The persistent activation even months after injury stresses the need for long-term follow-up, since late phase events otherwise will be missed. Peripheral activation of brain recognizing T-cells may lead to an increased risk of autoreactive brain damage later in life at time points where the blood brain barrier permits entrance of T-lymphocytes *e.g.* infections. Since some neurodevelopmental disorders, such as autism spectrum disorders, have been linked to immune activation and dysregulation [39], [40], it is possible that neurodevelopmental impairments in children after HI encephalopathy could be partly explained by latent inflammation. Further studies are needed in order to clarify the role and interactions of innate and adaptive immunity after HI brain injury. Understanding the basic functions and temporal interactions of these cells will pave the road for new immunomodulatory therapeutic strategies and are a vital prerequisite for the optimal timing of such treatments.

## Supporting Information

Table S1 **Details on markers and target cell populations with antibodies used for flow cytometry.**
(DOC)

Methods S1 (DOC)
