# Supplementary material for: Long Lasting Local and Systemic Inflammation after Cerebral Hypoxic ischemia in Newborn Mice
Source: PLoS One. 2012 May 2;7(5):e36422. doi: 10.1371/journal.pone.0036422 (PMC3342175; doi:10.1371/journal.pone.0036422)
Supplement: Table S1 — Details on markers and target cell populations with antibodies used for flow cytometry. (DOC) [file pone.0036422.s001.doc]

## Online supplement

# Long term self-reactive systemic inflammation after cerebral hypoxic ischemia

Max Winerdal MD^1*^, Malin Elisabeth Winerdal MD^2*^, Johan Kinn MD^2^, Vijay Urmaliya PhD^3^, Ola Winqvist MD, PhD^2**^ and Ulrika Ådén MD, PhD^1**^

^*^Both authors contributed equally. ^**^Shared last authorship.

^1^Department of Woman and Child Health, Karolinska Institutet, Sweden

^2^Department of Medicine, Unit of Translational Immunology, Karolinska Institutet, Stockholm, Sweden

^3^Department of Physiology and Pharmacology, Karolinska Institutet, Stockholm, Sweden

**Corresponding author:** Max Winerdal

Astrid Lindgren Children’s hospital Q2:07, Karolinska hospital, Stockholm, Sweden

Phone: +46 70 771 28 33

Fax: +46 8 517 773 53

E-mail address: [max.winerdal@ki.se](mailto:max.winerdal@ki.se)

## Supplemental methods

### Proliferation assay

Single-cell suspensions of splenocytes were prepared from unoperated, age-matched controls 3 and 5 months after HI as described for flow cytometry, and dissolved in RPMI-1640 with 10% BGS, 1% PeSt, 1% Glutamine, 50μM 2-ME, and 1% Pyruvate. 3-4x10^5^ cells per well were added to 96-well plates and stimulated with 10μg/mL Con A, brain homogenate or PBS. Brain homogenate was prepared from damaged/undamaged brain, homogenized in 5mL RPMI-1640, and kept at -20°C. For stimulation, brain homogenate was added at a ratio of 1:100. Plates were kept at 37°C for two to six days, pulsed with 1μCi/well [^3^H]Thymidine for 18h and frozen at -20°C. Plates were thawed and well content transferred to a glass fibre filter (Wallac) by a cell harvester (TOMTEC). For detection of radioactivity, Meltilex A – Melt on scintillation sheets (Wallac) were used and radioactivity was measured in a 1205 Betaplate Liquid Scintillation Counter (Wallac). Proliferation index was calculated as: cpm(brain homogenate)/cpm(PBS).
